# Mental health disorders and adolescent peer relationships

**DOI:** 10.1016/j.socscimed.2020.112973

**Published:** 2020-05

**Authors:** Emily Long, Maria Gardani, Mark McCann, Helen Sweeting, Mark Tranmer, Laurence Moore

**Affiliations:** a University of Glasgow, School of Social and Political Sciences, MRC/CSO Social & Public Health Sciences Unit, 200 Renfield Street, Glasgow, G2 3AX, United Kingdom; b University of Glasgow, School of Psychology, 62 Hillhead Street, Glasgow, G12 8QB, United Kingdom; c University of Glasgow, MRC/CSO Social & Public Health Sciences Unit, 200 Renfield Street, Glasgow, G2 3AX, United Kingdom; d University of Glasgow, School of Social and Political Sciences, Adam Smith Building, Bute Gardens, Glasgow, G12 8RT, United Kingdom

**Keywords:** Adolescents, Mental health, Social networks, Peers, Disruptive behaviour disorders, Anxiety disorders

## Abstract

**Rationale:**

Mental health disorders often arise during adolescence, with disruptive behavior disorders and anxiety disorders among the most common. Given the salience of peer relationships during adolescence, and research suggesting that mental health disorders negatively impact social functioning, this study uses novel methodology from social network analysis to uncover the social processes linking disruptive behavior disorders and anxiety disorders with adolescent friendships. In particular, the study focuses on peer withdrawal, peer popularity, and peer homophily in relation to both disorders.

**Methods:**

Data come from 15-year old students in four Scottish secondary schools (N = 602). Diagnoses of disruptive behavior disorders and anxiety disorders were produced using the Diagnostic Interview Schedule for Children, and peer relationship data were obtained through a friendship nomination survey. Exponential random graph models were used to estimate the probability of peer withdrawal, peer popularity, and peer homophily based on each disorder.

**Results:**

Results demonstrated that adolescents with disruptive behavior disorders were more popular than their peers without disruptive behavior disorders (OR: 1.47, CI: 1.20, 1.87). Friendship was also more likely between two adolescents both with or both without disruptive behavior disorders (OR: 1.26, CI: 1.07, 1.47), demonstrating peer homophily. There was no evidence that anxiety disorders were related to adolescent peer relationships.

**Conclusions:**

Findings from this study suggest that disruptive behavior disorders may be socially rewarded (e.g., peer popularity) and socially clustered (e.g., homophily), whereas anxiety disorders show no such trends. Thus, intervention efforts must account for the peer social status that may be gained from engaging in disruptive behavior during this developmental period. Further, given that similarity in DBD status is associated with an increased likelihood of friendship, adolescents are likely to be surrounded by peers who reinforce their behaviors.

## Highlights

• Peer relationships are associated with mental health disorders during adolescence.
• Adolescents with disruptive behavior disorders experience increased peer popularity.
• Homophily on disruptive behavior disorders is linked to friendship.
• Anxiety disorders are not related to adolescent social connections.

## 1 Introduction

Adolescence marks a particularly vulnerable period for the onset of poor mental health. Globally, between 10 and 20% of adolescents experience mental health disorders, and approximately half of all mental health disorders first emerge before the age of 14 (WHO, 2018). Of these disorders, disruptive behavior disorders (DBD) and anxiety disorders (AD) are among the most common (Ogundele, 2018). DBD and AD are linked with continued poor mental health in adulthood (Kessler et al., 2007), and associated with deficits in multiple domains during adolescence, including social functioning (Kingery et al., 2010; Milledge et al., 2019; Mrug et al., 2012).

This is particularly problematic in that adolescence is characterized by a heightened salience of relationships with peers (Crosnoe and Johnson, 2011; Rubin et al., 2006), and a lack of friends is associated with increased depression and decreased self-worth (Prinstein and Dodge, 2008; Rubin et al., 2006). Further, adolescent mental health is increasingly recognized as a priority area for public health policy (House of Commons, 2019; World Health Organization, 2013). As such, in order for research to effectively inform the design of policy programs aimed at enhancing the wellbeing of this vulnerable population, it is critically important to elucidate the interconnections between mental health disorders and adolescent peer relationships.

Recent advances in the field of social network analysis offer a powerful framework to address such questions. Based on social network theory (Kadushin, 2012; Valente and Pitts, 2017), social network methods embed individuals within their larger social environment in order to identify patterns of social connections. When applied to an adolescent context, social network methods can be used to examine peer relationships (e.g., friendships) within a school (i.e., typical social network), and estimate the extent to which individual attributes are related to the observed social structure. Previous research using these methods has shown that peer relationships form around important health indicators, such that adolescents who are obese are socially marginalized or isolated from their peers (Schaefer and Simpkins, 2014), while adolescents who use substances are often popular (Ali et al., 2014).

The key advantage of social network methods over traditional statistical approaches (e.g., regression models) is the ability to measure the association of individual attributes (e.g., DBD and AD) with social outcomes, while also controlling for the social network itself (Snijders, 2001; O’Malley, 2012). For example, endogenous properties of social networks (e.g., the way individuals within a social network are connected) are known to impact social structure, such that relationships (e.g., friendships) are more likely between individuals with shared social connections (Steglich et al., 2010). Similarly, social ties are more likely between individuals with shared sociodemographic attributes, such as gender and ethnicity (McPherson et al., 2001). Social network methods explicitly account for these structures when measuring the extent to which individual characteristics (e.g., DBD and AD) are related to social connections. Thus, given the importance of friendships during adolescence (Crosnoe and Johnson, 2011; Rubin et al., 2006), and research suggesting that mental health disorders impact social functioning (Kingery et al., 2010; Milledge et al., 2019; Mrug et al., 2012), social network methods are uniquely situated to uncover the underlying peer processes surrounding adolescent DBD and AD. Consequently, the current study employs methods from social network analysis to measure the extent to which DBD and AD are related to patterns in adolescent friendship structure within four secondary schools in Scotland.

### 1.1 The social context of adolescent mental health disorders

DBD and AD represent two markedly different classifications of mental health disorders. DBD include diagnoses of attention deficit disorder, conduct disorder, and oppositional defiant disorder (American Psychiatric Association, 2000). AD include diagnoses of social phobia, separation anxiety, specific phobia, panic attacks, agoraphobia, generalized anxiety, selective mutism, obsessive compulsive disorder, and post-traumatic stress disorder (American Psychiatric Association, 2000). Symptoms of DBD tend to be external and visible (e.g., questioning rules, low frustration tolerance, impulsiveness) while AD manifest in a more internal and concealed manner (e.g., feeling nervous, obsessive thoughts; American Psychiatric Association, 2000).

Previous research suggests that adolescents with DBD experience elevated levels of peer conflict and peer rejection (Fabiano et al., 2010; Milledge et al., 2019; Mrug et al., 2012), though these studies tend to rely on teacher- and parent-reported peer difficulties (Fabiano et al., 2010), which may differ from adolescent self-report (Arman et al., 2013; Roy et al., 2010). The impact of AD on adolescent peer relationships is less clear, with some research suggesting that adolescents with AD have significantly fewer friends than their non-AD peers, yet comparable friendship quality (Scharfstein et al., 2011), while other studies demonstrate a significant relationship between anxiety symptoms and experiences of peer rejection and victimization (Kingery et al., 2010).

Although social network studies have risen in popularity in recent years (Valente and Pitts, 2017), no study to date has used these methods to specifically examine adolescent DBD and AD. That said, social network studies of general mental wellbeing (e.g., emotional and behavioral symptoms) have found mental health-related clustering within friendship groups (i.e., peer homophily), such that adolescents with poor mental health tend to be friends with peers who also have poor mental health (Baggio et al., 2017). In addition, social network studies of non-clinical antisocial or externalizing behavior (e.g., rule-breaking, substance use) have found these behaviors to be associated with peer popularity (Franken et al., 2017; Sandstrom and Cillessen, 2006), while studies of adolescent depression demonstrate associations with social withdrawal from peers (Pachucki et al., 2015; Schaefer et al., 2011).

Taken together, research suggests that adolescent DBD and AD have implications for social relationships, and mental health more broadly is related to patterns of social network structure (e.g., peer popularity, peer withdrawal, peer homophily). Given the unique ability of social network methods to objectively quantify adolescent friendship patterns, the current study extends previous research by using these methods to determine the extent to which DBD and AD are associated with adolescent peer popularity, withdrawal, and homophily.

To accomplish this, the study makes use of sociometric data, wherein adolescents are asked to identify individuals from their school who they consider to be a friend. These nominations can be decomposed into incoming and outgoing friendship ties, thereby allowing for an examination of the peer processes linking DBD and AD and friendship. First, peer popularity of adolescents with DBD and AD can be tested by comparing the likelihood of friendship ties directed toward adolescents with DBD or AD versus ties directed toward adolescents without DBD or AD. Second, peer withdrawal of adolescents with DBD and AD can be tested by comparing differences in the likelihood of sending friendship nominations according to whether the adolescent has DBD or AD. Lastly, homophily, or similarity in DBD or AD, can be tested in order to determine if friendships are more likely between two adolescents with similar mental health status.

By specifically examining the associations between peer popularity, peer withdrawal, and peer homophily in relation to DBD and AD, the study offers novel insight into the role of mental health disorders in adolescent social relationships. Given the use of schools as intervention settings for programs targeting health behavior (Stigler et al., 2011), a clearly delineated understanding of the extent to which DBD and AD are associated with school-based friendships is necessary for effective program design. For example, adolescents with AD may be particularly withdrawn or unpopular with their peers, suggesting intervention strategies based on the development of social connections. Conversely, peer popularity based on DBD would suggest that adolescents with DBD may be reinforced by their social status. As such, the study asks the following research questions:

1. Do adolescents with (a) DBD and (b) AD have a different probability of receiving friendship nominations (representing peer popularity) than adolescents without these disorders?
2. Do adolescents with (a) DBD and (b) AD have a different probability of sending friendship nominations (representing peer withdrawal) than adolescents without these disorders?
3. Is there a higher probability of friendship between two adolescents both with or both without (a) DBD or (b) AD (representing peer homophily)?

## 2 Method

### 2.1 Study design and participants

Data for this study come from 15 to 16 year old students in four secondary schools (*N* = 602), participating in a larger study of 22 schools (*N* = 3194 students; Sweeting et al., 2008). The study was conducted in 2006 within a heterogeneous urban area around Glasgow in the West of Scotland. The aim of the larger study was to investigate the links between students’ peer group status and levels of stress, and the relationships of these with mental health and health behaviors (e.g. Kelly et al., 2008; West et al., 2010). Students in all 22 schools completed a questionnaire and took part in a brief interview with trained survey assistants. In the four-school sub-sample which forms the basis of the analyses presented here, students also completed a computerized DSM-IV compatible (American Psychiatric Association, 2000), audio version of the Diagnostic Interview Schedule for Children (Costello et al., 1984), the “Voice-DISC” (Shaffer et al., 2000; West et al., 2003) within a day of the larger study data collection.

Whereas the full sample of 22 schools provides data on general mental wellbeing and social relationships, data on clinically diagnosable mental health disorders is restricted to the four school sub-sample, and thus these schools serve as the current sample. Schools in the Voice-DISC sub-sample differed from the larger study sample in respect to a higher survey response, lower proportion of males, and lower mean deprivation score (Sweeting et al., 2008). The study used a saturated design, such that all students within the grade-level at each school were asked to participate. Average response rate for the questionnaire across the four schools was approximately 81%. Within the four schools in the Voice-DISC sub-sample, 86% of those who had completed a questionnaire also completed a Voice-DISC. Permission for all study components was received from the University of Glasgow Social Science Faculty Ethics Committee (SSL/05/03). Informed consent was obtained from adolescents and their parents.

### 2.2 Measures

#### 2.2.1 Peer relationships

Peer relationships were measured through sociometric data asking adolescents to “write the names of up to six friends”. Adolescents indicated whether each friend was: in the same school year; a higher year; a lower year; a different school; or had left school. Given that data were only collected on students within a single year group, friendship ties in the current study were restricted to those in this year group. ID codes were used to link adolescents to their nominated friends, allowing for the formation of year group-level networks. By using a saturated sample design in which all students within the grade-level were surveyed, the study provides whole-network data (e.g., all individuals within a naturally-bounded network), a requirement of the analyses (Robins et al., 2007).

#### 2.2.2 Mental health disorders

DBD and AD were measured using the Voice-DISC, a replica of the interviewer version of the DISC with evidence suggesting it is at least as reliable (Lucas, 2003). Adolescents self-administered the Voice-DISC using laptop computers in groups of up to 40, using headphones to listen to questions read out by an interviewer and answering via the keyboard, with the direction and content of the interview depending on responses to specific questions (Shaffer et al., 2000). Further details are available (West et al., 2000). Based on the DSM-IV, disorders were grouped into two types of mental health disorders; DBD and AD. Though newer versions of the DSM have been released since the time of data collection for this study (American Psychiatric Association, 2013), the Voice-DISC instrument in the current study was tested against DSM-IV criteria, and thus the DSM-IV categories of DBD and AD are used. DBD consisted of attention-deficit hyperactivity disorder, conduct disorder, and oppositional defiant disorder. AD consisted of social phobia, separation anxiety, specific phobia, panic attacks, agoraphobia, generalized anxiety, selective mutism, obsessive compulsive disorder, and post-traumatic stress disorder. Binary variables for both DBD and AD were created, such that adolescents with sub-clinical scores on all diagnoses were given a score of 0, and adolescents who met the clinical threshold for at least one diagnosis were given a score of 1 in the respective disorder group (i.e., DBD or AD).

#### 2.2.3 Peer popularity, peer withdrawal, and peer homophily

Unique to social network studies, all variables related to network structure and position are based on friendship nominations, rather than administered scales. Thus, the measures of peer popularity, peer withdrawal, and peer homophily were derived from friendship nominations and specified using the “ERGM” package within the R Language and Environment for Statistical Computing. DBD and AD indegree, parameterized using the ‘nodeifactor’ effect, measures the extent to which DBD and AD affect the likelihood of being nominated as a friend by others. This represents the desirability, or popularity, of adolescents with DBD or AD. DBD and AD outdegree, parameterized using the ‘nodeofactor’ effect, measures how DBD and AD affect the likelihood of nominating others as friends. This represents reaching out for friendships, or level of sociability in the network. Thus, a significant and negative coefficient would indicate that adolescents with DBD or AD are more socially withdrawn in comparison to adolescents without DBD or AD. Peer homophily related to DBD and AD was measured using the ‘nodematch’ effect, which calculates the likelihood of friendship based on whether both friendship sender and receiver meet criteria for DBD or AD. Given the cross-sectional nature of the study, the variables of peer popularity, withdrawal, and homophily, in addition to all variables in the study, are measured statically, and do not represent an across-time process.

#### 2.2.4 Covariates

In order to provide accurate estimates of associations between DBD and AD and adolescent friendships, other factors potentially related to social connections must be controlled (Steglich et al., 2010; Veenstra and Steglich, 2012). Ethnicity was reported in the brief interview (“Which of these groups best describes you?”) based on 2001 UK census categories, split, for the purpose of analysis into a binary variable indicating White versus all other ethnicities. All other covariates were reported in the questionnaire. Family affluence (Currie et al., 2008) was derived via items in respect of: number of family cars, vans or trucks; having own (not shared) bedroom; number of family computers; and number of family holidays in the past year, resulting in a variable ranging from 0 to 7 (α = 0.65). Consistent with previous research (Due et al., 2009), the 8-point scale was recoded to reflect a 3-point variable of low (0–3), medium (4–5), and high (6–7) affluence. Gender was a binary variable (male/female). Age was calculated to the nearest month, based on date of birth and date of survey. Ethnicity and age were dropped from the final models due to low levels of variability in the data. Given that adolescent peer relationships are also known to form around shared substance use patterns (Long et al., 2017; Wang et al., 2016), use of alcohol and cigarettes were included as covariates. Alcohol use was based on responses to “How often do you have an alcoholic drink (not just a sip)”, resulting in a 4-point variable, ranging from never to every day. Smoking was measured with a 3-point scale, including never at all, tried once/used to but gave up/occasionally, and regularly (one or more cigarettes a week).

#### 2.2.5 Missing data

Rates of missing data were relatively low; approximately 1% on covariates and 14% on the mental health diagnoses. Multiple imputation using chained equations (Raghunathan et al., 2001) was used to impute missing values for covariates and mental health variables. Imputation methods were not used for social network ties given that: 1) methods for treating missingness in social network ties remain underdeveloped (Huisman and Krause, 2017), and 2) only two students from the entire sample did not provide friendship nomination data (e.g., no outgoing ties). Imputation for all covariate and mental health data was carried out in the Mice package (van Buuren and Groothuis-Oudshoorn, 2011) in R, with results of analyses conducted on multiply-imputed datasets pooled using Rubin's Rules (Rubin, 1987). Goodness of fit diagnostics were conducted on all imputations, such that imputed values were compared to observed values using a kernel density estimator (van Buuren and Groothuis-Oudshoorn, 2011). Imputations were further assessed by calculating the between-imputation variance in model results (van Buuren and Groothuis-Oudshoorn, 2011) to ensure consistency across imputed datasets.

### 2.3 Analyses

Exponential random graph models (ERGMs; Robins, Pattison, Kalish and Lusher, 2007) were used to investigate the relationship between DBD and AD and adolescent friendship connections. ERGMs are probabilistic statistical models that assess the extent to which the presence of a network tie (e.g. a friendship nomination in a social network) is related to individual, dyadic, and structural features of the network (i.e., the way individuals within a social network are connected). Unlike traditional statistical methods, the ERGM is capable of studying how DBD and AD relate to friendship ties, while also accounting for endogenous properties of social networks which also affect friendships (Snijders, 2001; O’Malley, 2012). For example, reciprocity (e.g., mutuality in friendship nominations) and transitivity (e.g., friendship based around common others) contribute to the likelihood of friendship ties (Snijders, 2001; Van de Bunt et al., 1999) and are accounted for in the models (Robins et al., 2007).

Parameters identified a priori based on previous research (e.g., gender, ethnicity; McPherson et al., 2001) and theoretically-driven model parameters (e.g., peer popularity) are estimated in order to determine the attributes and social processes that most likely generated the observed peer relationships in each school. Markov chain Monte Carlo maximum likelihood estimation (MCMCMLE) is used to test model parameters. The results of the ERGM can be interpreted in a similar fashion to logistic regression, thus increasing accessibility to social scientists. For example, the model coefficients can be exponentiated to represent the log odds of a friendship tie conditional on all other ties within the network. Detailed information regarding model fitting and estimation of ERGMs can be found in Robins et al. (2007) and Hunter et al. (2008).

#### 2.3.1 Modeling procedure

The current study utilized the following forward-model building procedure. First, reduced models using only endogenous network effects (i.e., structural properties of the network itself) were fit to each sample school. Next, demographic and behavioral covariates were added to the models, before including DBD and AD variables separately. Lastly, models were run in which the three parameters of interest, peer popularity, peer withdrawal, and peer homophily, were included for DBD and AD simultaneously.

For all models, goodness of fit diagnostics were tested using Akaike information criterion (AIC; Akaike, 1973) and Bayesian information criterion (BIC; Raftery, 1995), in addition to visual plots of model fit produced by the modeling software. In this way, nested models were compared to determine the best fitting model specification, and model results were compared to target statistics to further ensure fit. In addition, a recently developed test for collinearity in model terms (Duxbury, 2018) was conducted, with the recommended variance inflation factor of 20 used as a cutoff. Detailed information regarding model fit and goodness of fit testing in ERGMs in available in Handcock et al. (2019). The list of parameters retained in the final models, along with their substantive interpretation, is provided in Table 1. All confidence intervals (*CI*s) are estimated at the 95% level).

**Table 1:** Interpretation of parameters in final models.

Parameter	Interpretation
Friendship Network Dynamics	
Edges	Number of friendship ties within the network
Mutual	Likelihood of reciprocated friendship
Two-path	Tendency of adolescents to become friends with adolescents their friends are already connected to
GW^a^ indegree	Tendency for some individuals to receive many nominations
GW^a^ outdegree	Tendency for some individuals to send many nominations
GW^a^ edgewise shared partner	Likelihood of friendship based on the number of friendship partners linking two individuals
Gender homophily	Likelihood of friendship between two individuals of the same gender
Ethnic similarity	Tendency to become friends with individuals of your same ethnicity
Smoking behavior	Main effect of smoking on total number of friendship ties
Drinking behavior	Main effect of drinking on total number of friendship ties
DBD^b^ indegree (peer popularity)	Difference in the likelihood of receiving friendship nominations based on whether the individual has DBD
DBD^b^ outdegree (peer withdrawal)	Difference in the likelihood of sending friendship nominations based on whether the individual has DBD
DBD^b^ homophily (peer homophily)	Likelihood of friendship based on whether both friendship sender and receiver have DBD
AD^c^ indegree (peer popularity)	Difference in the likelihood of receiving friendship nominations based on whether the individual has AD
AD^c^ outdegree (peer withdrawal)	Difference in the likelihood of sending friendship nominations based on whether the individual has AD
AD^c^ homophily (peer homophily)	Likelihood of friendship based on whether both friendship sender and receiver have AD

a Geometrically-weighted.

b Disruptive behavior disorder.

c Anxiety disorder.

Two supplemental sets of analyses were also conducted: 1) sensitivity analyses and 2) gender interaction models. Sensitivity tests were used to determine if the inclusion or exclusion of diagnoses within DBD or AD substantially altered the results. For example, the diagnosis of ADHD was removed from the DBD variable to test if its exclusion altered model results. Gender interactions for the three theoretically important parameters (i.e., peer popularity, peer withdrawal, and peer homophily) were tested to determine if there were differences between males and females in the extent to which DBD or AD were related to friendship. In all models, analyses were conducted on each school separately and then combined via random effects meta-analysis (Snijders and Baerveldt, 2003) using the ‘metafor’ package (Viechtbauer, 2010) in R Language and Environment for Statistical Computing.

## 3 Results

### 3.1 Descriptive statistics

Table 2 provides descriptive statistics of the sample. Adolescents were 15.4 years of age on average, 48% were male, and 88% reported White ethnicity. Approximately 8% met criteria for DBD, while approximately 18% met criteria for AD. Prevalence for each specific diagnosis is provided within Table 2. On average, adolescents provided 3.8 outgoing friendship nominations, and received 3.8 incoming nominations. Variability in descriptive statistics across the sample schools was tested using chi-square tests for categorical variables, and ANOVAs for continuous variables. Between-school variability in specific diagnoses was found, as well as variation across the sample in the prevalence of DBD and AD. In particular, School 4 showed high rates of DBD, while School 1 exhibited high rates of AD.

**Table 2:** Descriptive statistics of sample.

Characteristic	School 1 *n* = 144	School 2 *n* = 124	School 3 *n* = 201	School 4 *n* = 133	Aggregate *N* = 602	Difference between schools (*p*)
Male	47.9%	49.2%	48.3%	47.4%	48.2%	0.99
Age, mean (*SD*)	15.41 (0.3)	15.39 (0.3)	15.41 (0.3)	15.5 (0.3)	15.4 (0.3)	<0.001
White ethnicity	86.8%	96.0%	87.6%	82.7%	88.0%	<0.001
Family affluence, mean (*SD*)	2.19 (0.7)	2.08 (0.7)	2.49 (0.6)	2.25 (0.7)	2.3 (0.7)	<0.001
Disruptive behaviour disorders						
Oppositional defiant disorder (ODD)	<1%	4.8%	2.5%	5.3%	3.2%	0.11
Conduct disorder (CD)	4.2%	<1%	1.5%	6.7%	3.2%	<0.05
Attention deficit hyperactive disorder (ADHD)	<1%	0%	0%	3.7%	1.0%	<0.01
Anxiety disorders						
Social phobia	7.6%	4.0%	1.5%	3.8%	4.0%	0.05
Separation anxiety	5.6%	3.2%	1.0%	6.7%	3.8%	<0.05
Specific phobia	9.0%	4.0%	4.5%	9.0%	6.5%	0.18
Panic attacks	0%	<1%	<1%	1.5%	<1%	0.49
Agoraphobia	5.6%	4.8%	<1%	2.2%	3.0%	<0.05
Generalized anxiety	0%	<1%	<1%	1.5%	<1%	0.49
Selective mutism	<1%	<1%	<1%	0%	<1%	0.80
Obsessive compulsive disorder (OCD)	2.8%	6.5%	5.9%	5.3%	5.1%	0.45
Post traumatic stress disorder (PTSD)	0%	<1%	0%	<1%	0.3%	0.46
Binary disruptive behaviour variable	5.6%	5.7%	4.0%	14.3%	8.1%	<0.01
Binary anxiety variable	23.6%	15.3%	10.9%	15.8%	18.5%	0.03
Friendship indegree, mean (*SD*)	2.8 (2.1)	3.7 (2.4)	4.2 (2.7)	4.3 (2.6)	3.8 (2.5)	<0.001
Friendship outdegree	2.8 (1.6)	3.8 (1.7)	4.1 (1.6)	4.4 (1.7)	3.8 (1.8)	<0.001

*Note. N* = 602.

Between school differences were tested with chi-square tests for categorical variables, and one-way ANOVAs for continuous variables.

### 3.2 Peer relationships

Results for the final ERGMs are provided in Table 3. As anticipated, peer relationships followed basic endogenous properties of social networks, such as engaging in reciprocated friendship (i.e., mutual; *b* = 3.07, *CI*: 2.87, 3.27) and friendship based around common others (i.e., GWESP; *b* = 1.63, *CI*: 1.44, 1.83). Basic demographic characteristics were also associated with friendship patterns, with friendship between adolescents of the same gender more likely than cross-gender friendships (i.e., gender homophily; *b* = 0.80, *CI*: 0.61,0.99). In addition, substance use was associated with friendship, such that adolescents who smoked had a lower probability of friendship ties (i.e., smoking; *b* = −0.08, *CI* = −0.12, −0.04), and adolescents who drank alcohol had a higher probability of friendship ties (i.e., drinking; *b* = 0.08, *CI* = 0.04, 0.11). Parameter estimates were largely consistent across the sample, apart from significant variability in several endogenous properties (i.e., edges, GWESP), a common feature of social networks (Snijders, 2001; McPherson et al., 2001).

**Table 3:** Results from final models.

	Aggregate Estimate	Between-school variance	School 1	School 2	School 3	School 4
Parameter	Estimate*, CI*		Estimate, *CI*	Estimate, *CI*	Estimate, *CI*	Estimate, *CI*
Edges	−5.21 [-5.94, −4.48]	<0.05	−5.47 [-6.38, −4.56]	−5.21 [-6.28, −4.56]	−5.92[-6.72, −5.13]	−4.13 [-5.01, −3.62]
Mutual	3.07 [2.87, 3.27]	0.43	3.09 [2.59, 3.60]	2.78 [2.33, 3.23]	3.06 [2.71, 3.40]	3.28 [2.90, 3.67]
Two-path	−0.19 [-0.22, −0.15]	0.15	−0.27 [-0.34, −0.19]	−0.18 [-0.25, −0.12]	−0.16 [-0.21, −0.12]	−0.18 [-0.23, −0.13]
GW^a^ indegree	−0.55 [-0.83, −0.28]	0.84	−0.45 [-1.06, 0.16]	−0.37[-0.99, 0.25]	−0.61 [-1.09, −0.14]	−0.72 [-1.30, −0.15]
GW^a^ outdegree	0.98 [0.46, 1.49]	0.17	0.65 [-0.08, 1.37]	0.96 [0.12, 1.80]	1.78 [0.93, 2.62]	0.62 [-0.18, 1.41]
GWESP^b^	1.63 [1.44, 1.83]	<0.001	1.73 [1.56, 1.90]	1.74 [1.58, 1.91]	1.73 [1.61, 1.85]	1.34 [1.21, 1.47]
Gender homophily	0.80 [0.61, 0.99]	<0.05	0.74 [0.53, 0.96]	0.69 [0.49, 0.88]	1.14 [0.87, 1.41]	0.70 [0.53, 0.86]
Smoking	−0.08 [-0.12, −0.04]	0.26	−0.13 [-0.23, −0.02]	−0.03 [-0.12, 0.05]	−0.12 [-0.18, −0.05]	−0.05 [-0.12, 0.02]
Drinking	0.08 [0.04, 0.11]	0.21	0.17 [0.08, 0.27]	0.06 [-0.01, 0.13]	0.07 [0.02, 0.12]	0.05 [-0.02, 0.12]
Disruptive behavior indegree	0.39 [0.18, 0.59]	0.80	0.19 [-0.46, 0,85]	0.10 [-0.69, 0.88]	0.42 [ −0.07, 0.90]	0.44 [0.18, 0.69]
Disruptive behavior outdegree	−0.10 [-0.34, 0.14]	0.81	-.022 [-0.90, 0.46]	−0.44 [-1.25, 0.38]	−0.02 [-0.61, 0.56]	−0.04 [-0.35, 0.26]
Disruptive behavior homophily	0.23 [0.07, 0.39]	0.60	0.48 [0.00, 0.97]	0.16 [-0.54, 0.85]	0.35 [-0.06, 0.76]	0.17 [-0.02, 0.36]
Anxiety indegree	−0.06 [-0.21, 0.09]	0.69	−0.10[-0.38, 0.17]	0.04 [-0.26, 0.35]	0.00 [-0.29, 0.29]	−0.21 [-0.54, 0.12]
Anxiety outdegree	−0.13 [-0.31, 0.05]	0.83	−0.11 [-0.43, 0.20]	−0.01 [-0.38, 0.39]	−0.19 [-0.60, 0.21]	−0.23 [-0.60, 0.14]
Anxiety homophily	0.04 [-0.09, 0.16]	0.62	0.08 [-0.12, 0.29]	0.12 [-0.12, 0.35]	−0.07 [-.0.36, 0.23]	−0.09 [-0.39, 0.21]

*Note.* Between-school variance tested with Cochran's Q-test.

a Geometrically-weighted.

b Geometrically-weighted edgewise shared partner.

In terms of the theoretical variables of interest, three parameters: peer popularity, peer withdrawal, and peer homophily, were tested for both DBD and AD. Results demonstrated that adolescents with DBD had a higher probability of receiving friendship nominations than their peers without DBD (i.e., DBD indegree; *b* = 0.39, *CI*: 0.18, 0.59), suggesting peer popularity. This effect was strongest in School 4, marginally significant in School 3, and showed positive trends across the full sample. Cochran's Q-test suggested no significant variability in estimates across the schools (p < 0.80). Homophily based on DBD was also found (i.e., DBD homophily; *b* = 0.23, *CI*: 0.07, 0.39), such that friendship was more likely between two adolescents with similar DBD status. This effect was strongest in School 1, though marginally significant in School 3 and School 4, and showed positive trends across the full sample. Cochran's Q-test suggested no significant heterogeneity in the estimates across the schools (p < 0.60). The current study found no evidence that AD was related to measures of peer popularity, peer withdrawal, or peer homophily.

Sensitivity analyses revealed stable model results, such that the inclusion or exclusion of individual diagnoses did not significantly alter the direction or magnitude of the coefficients at both the school and aggregate level. Lastly, the gender interaction models revealed no evidence of differences between males and females in the relationship between DBD and AD and friendship ties (e.g., peer popularity). Due to concerns over model parsimony and low variability in the data, these interactions were removed from the final models.

## 4 Discussion

The current study is the first known effort to investigate the associations between DBD and AD and the structure of adolescent friendship networks. Based on social network theory (Kadushin, 2012; Valente and Pitts, 2017) and using rigorous DSM diagnostic criteria, the study employs methods from social network analysis to measure the extent to which DBD and AD are related to peer popularity, peer withdrawal, and peer homophily. Given the use of schools as intervention settings for programs targeting health behaviors (Stigler et al., 2011), including mental health (see Paulus et al., 2016), and current policy efforts on youth mental health (House of Commons, 2019; World Health Organization, 2013), the findings have direct implications for the design of programs aimed at improving mental health.

Consistent with previous research (Burk et al., 2007; Snijders et al., 2007), the current study found that peer relationships follow basic social network properties, such as friendship reciprocation. In addition, adolescents of the same gender had a higher likelihood of friendship, and friendships were structured around substance use behaviors, such that friendship ties to others in their school year-group were lower among adolescents who smoked cigarettes and higher among those who drank alcohol more frequently.

The study found significant evidence that DBD was related to patterns in adolescent friendships. Specifically, an adolescent with DBD was 1.47 (exp(0.39) = *OR*: 1.47, *CI*: 1.20, 1.87) times more likely to receive a friendship nomination than an adolescent who did not meet criteria for DBD, holding all other effects constant (see Table 3). This effect was found to be strongest in School 4, which had the highest prevalence of DBD within the sample (see Table 2). This suggests that DBD is associated with greater peer popularity, perhaps particularly in school contexts in which rates of DBD are high. The findings are consistent with earlier social network research demonstrating peer popularity of adolescents who engage in non-clinical levels of antisocial or externalizing behavior (Franken et al., 2017; Sandstrom and Cillessen, 2006). Given that diagnostic criteria for DBD include symptoms related to defiance and rule-breaking (American Psychiatric Association, 2000), the similarity between the current results and earlier work is unsurprising. Relatedly, delinquency, including rule-breaking and defiance, tends to peak in middle adolescence (e.g., ages 14–15; Abderhalden and Evans, 2016), perhaps contributing to the social desirability of this behavior in the current sample. However, the current findings are in contrast to previous research demonstrating peer conflict and peer rejection related to DBD (Fabiano et al., 2010; Milledge et al., 2019; Mrug et al., 2012). This difference is likely due to the use of sociometric data (e.g., friendship nominations) in the current study, rather than teacher or parent reports of peer relationships in traditional research (Fabiano et al., 2010; Milledge et al., 2019; Mrug et al., 2012). Thus, findings from the current study suggest an important distinction between subjective measures of peer relationship quality, and objective patterns in friendship connections. As a result, future work should explore the associations between DBD, friendship quality, and social network structure. Similarly, an investigation of these processes over time would allow for research to detect differences across age, as well as the impact of DBD on changes to peer popularity over time. For example, though symptoms of DBD (e.g., rule-breaking) were socially rewarded in the current study, this may not hold true in younger adolescents where prevalence of delinquency is lower (Abderhalden and Evans, 2016).

Adolescent peer relationships were also structured around similarity, or homophily, on DBD. For example, adolescents were 1.26 times (exp(0.23) = *OR*: 1.26, *CI*: 1.07, 1.47) more likely to be friends with a peer with the same DBD status as themselves, holding all other effects constant (see Table 3). The findings were strongest in School 1, which had, on average, smaller friendship groups than the other schools (see Table 2). Together, this suggests that DBD is clustered within specific friendship groups, rather than randomly distributed throughout the social network, perhaps particularly in school environments where students engage in smaller friendship groups. The current findings are in line with previous research demonstrating similarity between friends in general mental wellbeing (e.g., emotional and behavioral symptoms; Baggio et al., 2017). Peer homophily on health has been demonstrated across a range of health attributes (Jeon and Goodson, 2015; Valente and Pitts, 2017), and the current study suggests that this preference extends to clinical measures of DBD. Though the current results provide evidence of peer homophily on DBD, limitations in the data (e.g., low prevalence of ties between DBD peers) preclude testing for differential homophily (Robins et al., 2007). Future research should aim to compare the extent to which non-DBD peers cluster together, versus the clustering of DBD peers.

In contrast to the findings for DBD, the study found no evidence that AD was related to adolescent peer relationships. To this end, the findings suggest that the social network structure of adolescents with AD does not differ significantly from their peers without AD. Whereas the symptom manifestation of DBD is likely to be external and visible to peers (e.g., low frustration tolerance; American Psychiatric Association, 2000), the symptoms of AD may be more discreet (e.g., obsessive thoughts; American Psychiatric Association, 2000), and thus less likely to impact social network structure. That said, the findings differ from previous research where AD was shown to be associated with peer rejection and victimization (Kingery et al., 2010), though the focus of the current study was on objective social network measures (e.g., peer popularity, peer withdrawal, peer homophily) of peer relationships, rather than the subjective nature of peer relationship quality. Thus, it is critical for future research to simultaneously examine relationship quality and peer social network structure in order to gain a nuanced understanding of the associations between AD and adolescent social relationships.

Together, findings from this study suggest that adolescent DBD may be socially rewarded (e.g., peer popularity) and socially clustered (e.g., peer homophily), whereas adolescent AD show no such trends. Moreover, sensitivity analyses revealed that the inclusion or exclusion of individual diagnoses did not significantly alter the direction or magnitude of the coefficients, thus indicating that the diagnoses within each category (DBD and AD) function similarly in terms of their impact on adolescent social relationships. As such, the study has important implications for the design of intervention efforts surrounding mental health, particularly for adolescent DBD. For example, intervention efforts must account for the peer social status that may be gained from engaging in disruptive behavior during this developmental period. Moreover, given that similarity in DBD was associated with an increased likelihood of friendship, the use of social network data collection (e.g., sociometric information on friendships) could be used to identify high-risk peer groups where symptoms of DBD may be clustered, and thus intervention efforts may be most needed.

The study also has implications for practitioners working with adolescents with DBD in highlighting that there may be distal social reasons for engaging in disruptive behavior not immediately evident in a clinical setting. Lastly, the findings suggest that school-based intervention efforts that focus only on students who are isolated or marginalized from their peers are likely to be unsuccessful in reaching students with AD, given that adolescents with AD were equally embedded in the peer network.

### 4.1 Limitations and conclusions

Several limitations of the current study need to be mentioned. Firstly, the study is cross-sectional in nature, precluding the use of longitudinal analyses capable of measuring peer processes over time; for example, the co-evolution of friendship ties and behavior. However, the use of cross-sectional methods does allow an examination of the extent to which DBD and AD were associated with adolescent peer relationship structure. Secondly, the study focuses on objective measures of friendship connections captured through a social network design, but does not include relationship quality measures. Thirdly, given the age of the data (i.e., collected in 2006), the diagnoses included in the disorder groups of DBD and AD reflect the categorizations and clinical criteria of the DSM-IV, rather than the more recent DSM-V. In addition, adolescents were restricted to six friendship nominations, which may not capture an adolescent's full friendship group. Though a limited nomination design is sufficient in most cases (Frederickson and Furnham, 1998; Marsden, 2014) the number of nominations may affect statistical power (Stadtfeld et al., 2018), and hence the peer effects in the present study may be underestimated. The study is also restricted to 15-year old adolescents within four schools in Scotland, thus limiting generalizability to a wider population of Scottish youth, or adolescents outside the UK context. Lastly, there may be systematic differences between adolescents who opted to not participate in the study and those who took part, a common feature of social survey designs.

Despite these limitations, the study advances research in several ways. It is the first known effort to apply methodology from social network analysis to the investigation of adolescent DBD and AD. By doing so, the study builds upon a body of research demonstrating social implications of adolescent mental health (Baggio et al., 2017; Kingery et al., 2010; Milledge et al., 2019; Mrug et al., 2012), but uniquely identifies the nature and extent to which DBD and AD are associated with patterns in peer relationships (e.g., peer popularity, peer withdrawal, peer homophily) in school-based friendship networks. DBD, in particular, was found to not only be related to peer popularity, but also a tendency of adolescents to engage in friendships with others who share their DBD status. Given the global scope of adolescent mental health disorders (WHO, 2018), and increasing recognition of mental health as a critical focus for public health policy (House of Commons, 2019; World Health Organization, 2013), this work provides new insight into the social context of adolescent DBD and AD.

## CRediT authorship contribution statement

**Emily Long:** Conceptualization, Methodology, Formal analysis, Writing - original draft. **Maria Gardani:** Conceptualization, Writing - review & editing. **Mark McCann:** Conceptualization, Methodology, Validation, Writing - review & editing. **Helen Sweeting:** Conceptualization, Methodology, Writing - review & editing. **Mark Tranmer:** Conceptualization, Methodology, Writing - review & editing. **Laurence Moore:** Conceptualization, Writing - review & editing.

## Declaration of competing interest

None.
