# Supplementary material for: Mental health disorders and adolescent peer relationships
Source: Soc Sci Med. 2020 May;253:112973. doi: 10.1016/j.socscimed.2020.112973 (PMC7248572; doi:10.1016/j.socscimed.2020.112973)
Supplement: Multimedia component 1 [file mmc1.pdf]

Appendix. R script for ERGM estimation, model diagnostics, and meta-analysis.

R script for final models.

```
set.seed(200)
results = list()
counter <- 0

schools <- list(unique(V(imp_data)$schoolid)) %>%
   data.frame %>%
   setNames(c("name"))

 for (i in schools$name) {
   temp <- induced_subgraph(imp_data, V(imp_data)$schoolid == i)
   temp <- asNetwork(temp)
   fit <-  ergm(temp ~ edges + mutual + twopath + gwidegree(1, fixed = TRUE) +
              gwodegree(1, fixed = TRUE) + gwesp(.5, fixed = TRUE) + + nodematch('sex') +
              nodecov('smoking') + nodecov('drinking') +
              nodeifactor('exbinary') + nodeofactor('exbinary') + nodematch('exbinary') +
              nodeifactor('anxbinary') + nodeofactor('anxbinary') + nodematch('anxbinary'),
            control=control.ergm(MCMC.burnin=5000,
                          MCMC.interval=1000, MCMC.samplesize=20000))
   name <- paste0("school_", i)
   prelist <- list()
   prelist$name <- name
   prelist$fit <- fit
   counter <- counter + 1
   results[[counter]] <- prelist
 }
```

R script for assessing model fit

```
# Example mcmc diagnostics for 7th model run
mcmc.diagnostics(results[[7]]$fit)

# Example model fit indices for 5th model run
par(mfrow=c(1,1))
example <- gof(results[[5]]$fit)
plot(example)

model1sim <- simulate(results[[5]]$fit, nsim=10)
```

R script for meta-analysis

```
# Example using School 1
# results 1, 5, 9, 13, 17 are all for school 1
School1 <- list(results[[1]], results[[5]], results[[9]], results[[13]], results[[17]],
           results[[21]], results[[25]], results[[29]], results[[33]], results[[37]]
)
```

```
# Make list of only s3 output
School1_output = list()
for (i in 1:length(school1)){
  school1_output[[i]] <- school1[[i]]$fit
}

# Pool the results
School1_pooled <- summary(pool(school1_output))

# Pool using scaler
# list of standard errors for edges
se = list()
for (i in 1:5) {
  se[[i]] <- summary(school1[[i]]$fit)$asyse['edges']
}

# list of estimates for edges
coef = list()
for (i in 1:5) {
  coef[[i]] <- as.numeric(coef(school2[[i]]$fit)['edges'])
}

# Unlist
vcoef <- unlist(coef)
vse <- unlist(se)

# Pool using Rubin's rules
impvar <- pool.scalar(vcoef, vse, n = nrow(nodes), k = 1)
impvar$qbar # pooled estimate
impvar$ubar # pooled se
impvar$b # between-imputation variance

# Combine output from all four schools
output <- list(school1_pooled, school2_pooled, school3_pooled, school4_pooled)

##Create a table with columns for param and se, and rows for each network
conf.all.coef.table <- matrix(NA, nr=4, nc = 2)

# Meta-analysis for 15th parameter
for (i in 1:length(output)){
  conf.all.coef.table[i,1] <- output[[i]]$estimate[15]
  conf.all.coef.table[i,2] <- output[[i]]$std.error[15]
}

conf.all.metareg <- rma(yi=conf.all.coef.table[,1],sei=conf.all.coef.table[,2],
                        slab=c("Sch1", "Sch2", "Sch3", "Sch4") , method = "REML")
```
